# Mitochondrial genome study of *Camellia oleifera* revealed the tandem conserved gene cluster of *nad5–nads* in evolution

**DOI:** 10.3389/fpls.2024.1396635

**Published:** 2024-09-03

**Authors:** Yiyang Gu, Liying Yang, Junqin Zhou, Zhun Xiao, Mengqi Lu, Yanling Zeng, Xiaofeng Tan

**Affiliations:** ^1^ Key Laboratory of Cultivation and Protection for Non-Wood Forest Trees, Ministry of Education, Central South University of Forestry and Technology, Changsha, China; ^2^ Academy of Camellia Oil Tree, Central South University of Forestry and Technology, Changsha, China; ^3^ Hunan Horticulture Research Institute, Hunan Academy of Agricultural Sciences, Changsha, China; ^4^ College of Landscape Architecture, Central South University of Forestry and Technology, Changsha, China; ^5^ School of Foreign Languages, Changsha Social Work College, Changsha, China

**Keywords:** *Camellia oleifera*, mitochondrial genome, Camellia, gene cluster, nad5-nads tandem genes

## Abstract

*Camellia oleifera* is a kind of high-quality oil supply species. Its seeds contain rich unsaturated fatty acids and antioxidant active ingredients, which is a kind of high-quality edible oil. In this study, we used bioinformatics methods to decipher a hexaploid Camellia oil tree’s mitochondrial (mt) genome based on second-generation sequencing data. A 709,596 bp circular map of *C. oleifera* mt genome was found for the first time. And 74 genes were annotated in the whole genome. Mt genomes of *C. oleifera* and three Theaceae species had regions with high similarity, including gene composition and gene sequence. At the same time, five conserved gene pairs were found in 20 species. In all of the mt genomes, most of *nad* genes existed in tandem pairs. In addition, the species classification result, which, according to the gene differences in tandem with nad5 genes, was consistent with the phylogenetic tree. These initial results provide a valuable basis for the further researches of *Camellia oleifera* and a reference for the systematic evolution of plant mt genomes.

## 1 Introduction

*Camellia oleifera* Abel., originating in China, is a non-wood forest that blooms and harvests in winter. *C. oleifera* is a kind of high-quality woody edible oil tree in Theaceae, which is famous for its rich unsaturated fatty acids in fruit (Tan, 2013). *Camellia* oil tree not only contains unsaturated fatty acids up to 90% but also is rich in antioxidant active ingredients such as squalene, sterol, vitamin E and polyphenols (He and He, 2002). *C. oleifera* with *Elaeis guineensis* Jacq., *Olea europaea* L. and *Cocos nucifera* L. are known as the world’s four famous oil species. In recent years, in China, with the support of national policies, such as sustainable development, green development and targeted poverty alleviation, the development of *C. oleifera* has been widely supported due to its high economic value, wide use, comprehensive development, and utilization potential. *C. oleifera* is called “iron crop” and “green oil reservoir” by farmers (Wu et al., 2018). Studies on the development characteristics of *C. oleifera* fruits (Zhang et al., 2021), self-incompatibility of flowers (Zhou et al., 2020; Lu et al., 2021), and other economic potential exploitation have enabled researchers to have a further understanding of *C. oleifera*. With continuous progress of various technologies, plant genome research has become one of the most popular research topics. For researchers, the genome is a reference book for understanding a species or an organelle. At present, the chloroplast genome of *C. oleifera* has been reported (Wu et al., 2020), and the nuclear genome is also being carried out gradually. *C. oleifera* mitochondrial (mt) genome will also be the focus of the next step.

Mitochondria are important organelles in eukaryotic cells, which are closely related to energy generation, fatty acid synthesis, and active protein synthesis (Niu et al., 2022). Mitochondria are semi-autonomous organelles that encode their own functional genes and are regulated by nuclear genes. The mt genome of plants is more complex than that of animals and single-celled eukaryotes. The mt genomes of plants have different structures, gene contents, and DNA mutation rate strategies to meet their specific needs for physical, photosynthetic, or physiological and biochemical functions (Cui et al., 2009). Concurrently, cytoplasmic male sterility and species evolution in plants are closely related to the mitochondria (Wang et al., 2020). More than 200 plant mt genomes have been published, and most of them are crop species. However, there are only two articles on mt genome of Theaceae plants (Zhang et al., 2019; Rawal et al., 2020). The mt genome sequence is not as conserved as the chloroplast genome sequence, but some gene families are also conserved in the mt genome. There may even be concatenation of two genes or a group of genes. In Niu’s study, two gene clusters (*rps12*-*nad3* and *rps3*-*rpl16*) were present in eight mitochondrial genomes (Niu et al., 2022). This is actually one of the dependencies on which all genomes can be assembled after sequencing.

With the development of sequencing technology, longer and more accurate reads can be obtained from the sequencing result, which enables many complex plant genomes to be gradually broken down. The sequencing results of one species include not only nuclear genome sequences but also mt genome sequences. Based on this, strategies and software extraction and assembly of mt genome sequences from whole-genome data are developing (King et al., 2014; Al-Nakeeb et al., 2017). MIA software (Green et al., 2008), for example, is an early mt genome assembly software. It takes mt genome sequences from the ancient human whole-genome sequence data and assembles them together. After then, software, such as MITObim (Hahn et al., 2013), ARC (Hunter et al., 2015), and NOVOPlasty (Dierckxsens et al., 2017), have been developed for every species other than people. Burns et al. (2015) also used MITObim to assemble the mt genome of *Cymbomonas tetramitiformis* when assembling its nuclear genome. Therefore, the genomes of complex species assembled from sequencing data can be used as a kind of data reference for research.

Therefore, the mt genome of *C. oleifera* was selected as the research object in this study. After obtaining all related reads from the sequencing data, the mt genome of *C. oleifera* was assembled by the MITObim software (v4.0.2) (Hahn et al., 2013). According to the sequence information of the genome, its structural characteristics and the situation of coding genes were annotated. The successful analysis of mt genome enables researchers to have a further understanding of *C. oleifera* and focus on the study of biological issues with more time and more energy.

## 2 Materials and methods

### 2.1 Materials and data background

The plant material *Camellia oleifera* cv. Huashuo was planted in the experimental field in Wangcheng district, Changsha, Hunan province (28°05′N, 113°2′E). Young leaves of adult *C. oleifera* trees were collected, preserved with liquid nitrogen, and sent to BGI Genomics for sequencing. The BGISEQ 500 Platform was used to build the second-generation sequencing database. The original data used in the experiment was extracted from the next-generation sequencing datasets of *Camellia oleifera*. All reads were trimmed and error corrected and then were provided for the assembly of mt genome of *C. oleifera* as data source.

### 2.2 Genome assembly

MITObim v4.0.2 (Hahn et al., 2013) was used for *C. oleifera* mt genome assembly. It can directly assemble the mt genomes of non-model species from DNA sequencing data embedded with MIRA and IMAGE modules. The mt genomes of *Camellia sinensis* var. *assamica* cv. Yunkang10 (YK10) (Zhang et al., 2019) and *Camellia sinensis* var. *assamica* cv. TV-1 (TV-1) (Rawal et al., 2020) were used as reference. All the paired-end sequencing reads of *C. oleifera* were mapped to the two mitochondrial reference sequences by BWA v0.7.12 (bwa index -p ref reference.fa) (Li and Durbin, 2009). According to the similarity of sequences, reads needed for mitochondrial assembly were captured preliminarily. Then, Samtools v1.9 was used to extract reads that were paired aligned to the reference sequence (bwa men *-t 16 ref.fa F.fq.gz R.fq.gz* | samtools view *-bF 12* | samtools sort *-@ 16 -m 1G -o output.bam*) (samtools view *-h output.bam*| tail *-n +4* | cut *-f 1*> map_reads.txt). All reads were integrated to a new sequence file. This file was used as the input file of MITObim to assemble the mt genome of *C. oleifera*. Concurrently, *Camellia sinensis* mitochondrial sequences (Zhang et al., 2019; Rawal et al., 2020) were used as the main reference sequence in the assembly process.

### 2.3 Genome annotation and visualization

The GeSeq (Tillich et al., 2017) tool was used to annotate the mt genome of *C. oleifera*. Protein-coding genes, transfer RNA (tRNA), and ribosomal RNA (rRNA) genes were annotated by BLAST with the existing plant mt genome data in NCBI database (https://www.blast.ncbi.nlm.nih.gov), including *Camellia sinensis* var. *assamica* cv. Yunkang10 (MK574876.1) (Zhang et al., 2019), *Camellia sinensis* var. *assamica* cv. TV-1 (NC_043914.1) (Rawal et al., 2020), *Vitis vinifera* (NC_012119.1) (Goremykin et al., 2009), *Triticum aestivum* (NC_036024.1) (Cui et al., 2009), *Oryza sativa* subsp. *indica* (NC_007886.1) (Tian et al., 2006), *Zea mays* subsp. *mays* (NC_007982.1) (Clifton et al., 2004), *Glycine max* (NC_020455.1) (Chang et al., 2013), *Gossypium arboreum* (NC_035073.1) (Chen et al., 2017), *Ziziphus jujuba* (NC_029809.1), *Bupleurum falcatum* (NC_035962.1), *Boechera stricta* (NC_042143.1) (Li et al., 2018), and others. Concurrently, tRNA genes in the mt genome were annotated again with tRNA scan-SE tool (Lowe and Eddy, 1997). The annotated genes with coverage and match less than 60% were manually eliminated, and the repeated annotation results were compared. The final annotation results were drawn by Draw Organelle Genome Maps (OGDRAW v1.3.1) tool (Greiner et al., 2019) with a circular map and a linear map.

### 2.4 Repeat analysis and RNA-editing site prediction

The repeat sequence detection in the mt genome of *C. oleifera* was carried out by MISA v2.1 (Beier et al., 2017) and REPuter tools (Kurtz et al., 2001). Simple repeats (SSR) were verified by MISA, with the minimum number of nucleotide repeats setting as 8, 4, 4, 3, 3, and 3 for monomer, dimer, trimer, tetramer, pentamer, and hexamer, respectively (Zhang et al., 2019). At the same time, incomplete repeats of SSR interrupted by a few bases (spacing less than 100 or equal to 100) were screened, identified, and located. Forward and palindromic repeats are confirmed by REPuter with a minimum length of 50 nt and a minimum fault tolerance of 8 nt. The RNA-editing site was predicted by the PREP-Mt web tool (Mower, 2005). By reading the location information of genes in the annotation file, the base sequences of protein-coding genes were obtained from the genome. The gene sequence file was adjusted according to the format required by the tool, and the threshold was set at 0.2.

### 2.5 Phylogenetic analysis

The phylogenetic tree was constructed by IQ-Tree software (Nguyen et al., 2015), carried by TBtools (Chen et al., 2020). Published mt genome data of 19 species were selected from the NCBI database, including 16 dicotyledons as follows: *Arabidopsis thaliana* (Ath, NC_037304.1) (Unseld et al., 1997), *Brassica napus* (Bna, NC_008285.1) (Handa, 2003), *Bupleurum falcatum* (Bfa, NC_035962.1), *Camellia gigantocarpa* (Cgi, OP270590) (Lu et al., 2022), *Camellia sinensis* (Csi, MK574876.1; NC_043914.1) (Zhang et al., 2019; Rawal et al., 2020), *Capsicum annuum* (Can, NC_024624.1), *Carica papaya* (Cpa, NC_012116.1) (Magee et al., 2010), *Glycine max* (Gma, NC_020455.1) (Chang et al., 2013), *Gossypium arboretum* (Gar, NC_035073.1) (Chen et al., 2017), *Gossypium barbadense* (Gba, NC_028254.1) (Tang et al., 2015), *Malus domestica* (Mdo, NC_018554.1) (Goremykin et al., 2012), *Nicotiana tabacum* (Nta, NC_006581.1) (Sugiyama et al., 2005), *Rhazya stricta* (Rst, NC_024293.1) (Park et al., 2014), *Spinacia oleracea* (Sol, NC_035618.1) (Cai et al., 2017), and *Vitis vinifera* (Vvi, NC_012119.1) (Goremykin et al., 2009), two monocots: *Cocos nucifera* (Cnu, NC_031696.1) and *Triticum aestivum* (Tae, NC_036024.1) (Cui et al., 2009), and one gymnosperm: *Ginkgo biloba* (Gbi, NC_027976.1). These include two published species of the genus *Camellia*. The annotation information of all species was compared manually, including the *Camellia oleifera*. The sequences of 15 conserved genes were extracted, respectively, and aligned using Muscle v5 (Edgar, 2021) with default parameters. After integrating the comparison results, the portable IQ-tree software carried by TBtools (Chen et al., 2020) was used to build the phylogenetic tree, setting the model parameter at Auto and Boostrap value at 1,000 (iqtree *-s TBtools5888937064767651616.tmpIn -pre supergene.fa -bb 1000 -bnni -m MFP -nt AUTO*).

### 2.6 Collinearity analysis

MCScanxX (Wang et al., 2012) was used to analyze collinearity among the mt genomes of four Theaceae species. The sequences and GFF (general feature format) annotation files of two *Camellia sinensis* mt genomes were downloaded from the NCBI database (MK574876.1;NC_043914. 1) and the data number of *Camellia gigantocarpa* is OP270590. The sequence files and annotation files were modified to meet the input file format of One Step MCScanX software in TBtools (Chen et al., 2020). Running the software with the parameters (*-CPU for BlastP 2 -E-value 1e-10 -Num of BlastHits 5*) obtained the collinearity analysis results of *C. oleifera* and the other three, respectively. Then, the resulting data files were used as input files of Dual Systeny Plot software to draw the colinear map of mt genomes.

## 3 Results

### 3.1 Assembly and annotation

**Figure 1:**
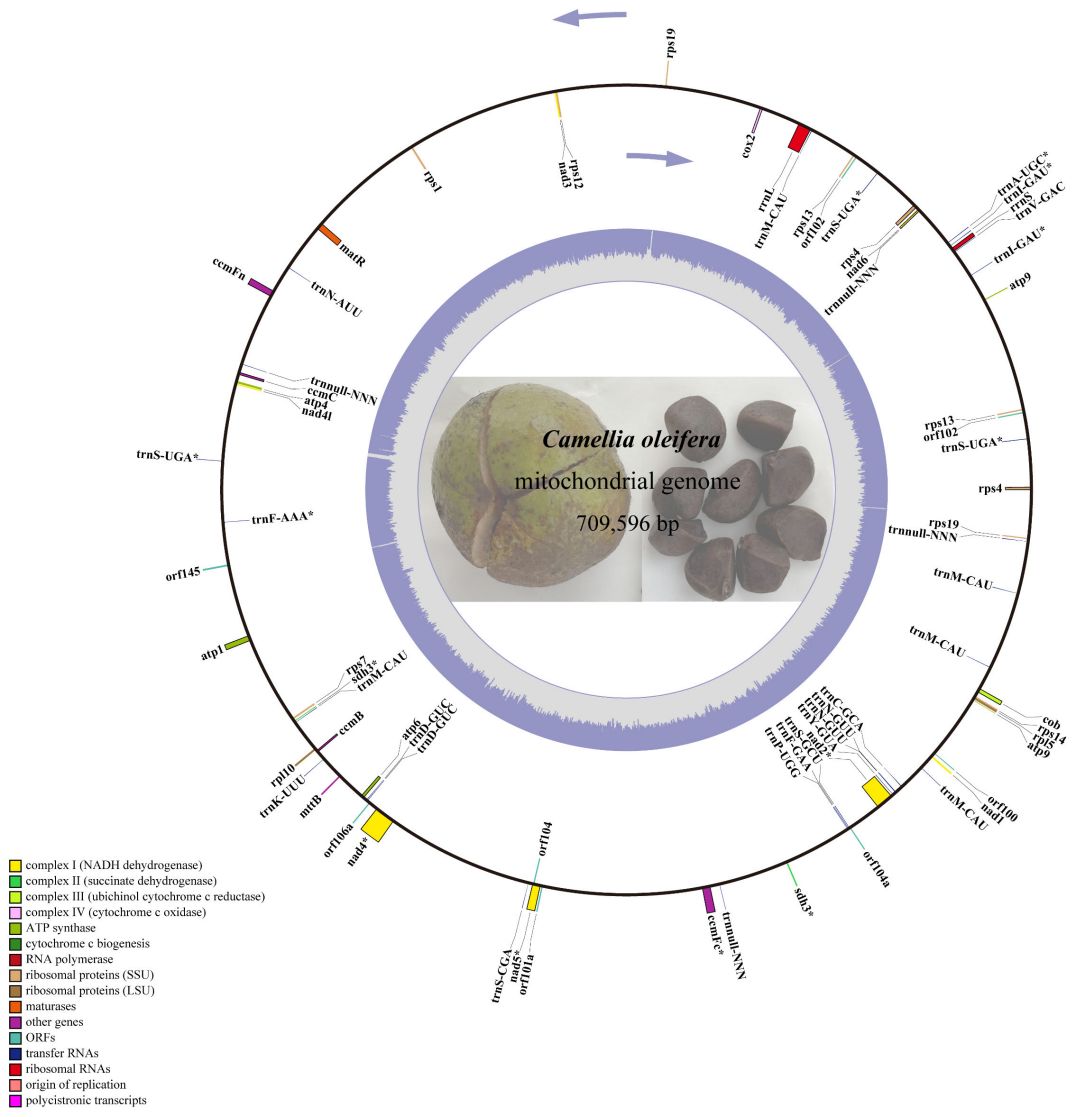
The circular map of *Camellia oleifera* cv. Huashuo mt genome. Gene map showing 74 annotated genes with different functional groups that are color-coded on the outer circle as transcribed clock-wise (outside) and transcribed counter clock-wise (inside). The inner circle indicates the GC content as gray purple plot.

The next-generation sequencing yielded 525 G data files, of which 34,720,712 reads were compared to the reference genome. A 709,596-bp circle mitochondrial map of *C. oleifera* was obtained, and GC content reached 45.33% (**Figure 1**). A total of 42 protein-coding genes (including *orf*), 29 tRNA genes, and 2 rRNA genes (*rrnL* and *rrnS*) were annotated in this mt genome. Among all protein-coding genes, Complex I (NADH dehydrogenase) family and Ribosomal protein (SSU) family exhibited the highest gene count. The total length of the exon (or CDS) region of the protein-encoding gene was 26,781 bp, including *sdh3*, *atp9*, *rps4*, *rps13*, *rps19*, and *orf102* with double copies, while the promoters of *nad2*, *rps4* (two copies), and *sdh3* were not “ATG.” Among 29 tRNA genes, there are five copies of *trnM-CAU*, four copies of *trnnull-NNN*, three copies of *trnS-UGA*, and two copies of *trnD-GUC*, *trnN-GUU*, and *trnl-GAU* (**Table 1**).

**Table 1:** Gene content of mt genome with three *Camellia* species.

Category		*Camellia oleifera* cv. Huashuo		*Camellia sinensis* var. *assamica*		*Camellia gigantocarpa*
				TV-1	YK10	
**Genome size (bp)**		709,309		707,441	701,719 + 177,329	970,410
**GC content (%)**		45.33		45.75	45.63/45.81	45.00
**Total genes number**		74		66	73	66
**Protein-coding** **genes number**	Complex I (NADH dehydrogenase)	35	*nad1*, nad2*, *nad3*, nad4*, *nad4l*, nad5*, *nad6*	31	44	44
	Complex II (succinate dehydrogenase)		sdh3*(3x)			
	Complex III (ubiquinol cytochrome *c* reductase)		*cob*			
	Complex IV (cytochrome *c* oxidase)		*cox2*			
	Complex V (ATP synthase)		*atp1*, *atp4*, *atp6*, *atp9* (2x)			
	Cytochrome c biogenesis		*ccmB*, *ccmC*, ccmFc*, *ccmFn*			
	Ribosomal proteins (SSU)		*rps1*, *rps4*(2x), *rps7*, *rps12*, *rps13*(2x), *rps14*, *rps19*(2x)			
	Ribosomal proteins (LSU)		*rpl5*, *rpl10*			
	Maturases		*matR*			
	Transport membrane proteins		*mttB*			
**Transfer RNAs**		29	trnA-UGC*, *trnC-GCA*, *trnD-GUC* (2x), trnF-AAA*, *trnF-GAA, trnK-UUU*, *trnM-CAU* (5x), *trnN-AUU*, *trnN-GUU* (2x), *trnP-UGG*, *trnS-CGA*, *trnS-GCU*, trnS-UGA* (3x), *trnV-GAC*, *trnY-GUA*, trnl-GAU* (2x), *trnnull-NNN* (4x)	29	24	19
**Other genes**		8	*orf100*, *orf101a*, *orf102 (2x)*, *orf104*, *orf104a*, *orf106a*, *orf145*	4	2	/
**Ribosomal RNAs**		2	*rrnL*, *rrnS*	2	3	3
**Total SSRs**		530		529	665	746
**RNA-editing sites**		413/35		429/31	478/44	483/44

*Gene with introns. Numbers of copies are shown in parenthesis for genes with multiple copies.

### 3.2 Repeat analysis

A total of 530 SSRs were identified in the mt genome of *C. oleifera*. Among them, monomer, dimer, trimer, tetramer, pentamer, and hexamer accounted for 33.2%, 44.7%, 4.7%, 14.9%, 2.1%, and 0.4%, respectively (**Table 2**). Among the 176 monomer repeats, A/T accounted for the main proportion, reaching 86.9%, and C/G was only 13.1%. Among the dimer repeats, AT repeats up to eight times and TA repeats up to nine times, and there were only two hexamers (CTATCC and TTTCTA) (**Supplementary Table 1**). Furthermore, a total of 50 pairs of long repeat sequence (repeat unit >50 bp) were identified, including 20 pairs of forward repeats and 30 pairs of palindromic repeats (**Table 3**). The length of the shortest repeats was 139 bp, and the longest was 10,565 bp (**Supplementary Table 2**). Moreover, forward repeats mainly cluster in the earlier part of the genome sequence, while reverse repeats cluster in the later part.

**Table 2:** Statistics of SSR motifs in the *Camellia oleifera* cv. Huashuo mt genomes.

SSR-motif		Repeats/number	SSR number	SSR %
**Monomer**	A	8/33, 9/30, 10/4, 11/2, 12/2, 13/1 72)	176	33.2
	C	8/5, 9/3 (8)		
	G	8/14, 9/1 (15)		
	T	8/41, 9/26, 10/8,11/2, 12/2, 15/1, 16/1 (81)		
**Dimer**	AC	4/5 (5)	237	44.7
	AG	4/41, 5/9, 6/1, 7/2 (53)		
	AT	4/13, 5/4, 6/3, 7/3, 8/1, 9/1 (25)		
	CA	4/1 (1)		
	CG	4/3 (3)		
	CT	4/32, 5/11, 6/2 (45)		
	GA	4/23, 5/9, 6/1 (33)		
	GC	4/2 (2)		
	GT	4/5, 5/1 (6)		
	TA	4/17, 5/6, 6/2 (26)		
	TC	4/26, 5/3, 6/2 (31)		
	TG	4/7 (7)		
**Trimer**	AAC, AAG, AGC, GCT, TAT, TTG	4/1 (6)	25	4.7
	ACT, CTT, GAA, TTC	4/2 (8)		
	TAA, TTA	4/3 (6)		
	TCT	4/4 (4)		
	GAT	5/1 (1)		
**Tetramer**	AACA, AACC, AAGC, AAGG, AATA, AATG, ACTA, AGCT, AGTC, ATAA, ATGG, CAAT, CAGA, CCAG, CCGA, CCTT, CGGG, CTAG, CTCC, GAAG, GAGC, GCTT, GGCC, GTGA, TAGA, TAGT, TGAA, TTAC, TTAT, TTCC, TTTG	3/1 (31)	79	14.9
	AAAT, ATAG, ATTC, CAAA, CTTC, CTTG, GAAA, GAAT, GATT, GGAA	3/2 (20)		
	AAGA	3/3 (3)		
	AAAG, AGAA, TCTT	3/4 (12)		
	TTCT	3/5 (5)		
	CTTT	3/6 (6)		
	CCTT, TATC	4/1 (1)		
**Pentamer**	AATTA, ACTAG, ATAGG, CTAAT, GAAAT, GCCTT, TAAAG, TTAAT, TTATA	3/1 (9)	11	2.1
	TAGAG	3/2 (2)		
**Hexamer**	CTATCC, TTTCTA	3/1 (2)	2	0.4

**Table 3:** Statistics of long repeat sequences in the *Camellia oleifera* cv. Huashuo mt genomes.

No.	Size (bp)	Palindromic Copy (P)		Size (bp)	Forward Copy (F)	
		Start 1	Start 2		Start 1	Start 2
**1**	174	17,713	364,406	722	0	87,394
**2**	162	54,865	684,866	582	727	88,125
**3**	191	105,254	648,172	433	1,332	88,732
**4**	281	152,801	709,308	447	1,764	89,165
**5**	236	153,082	709,073	139	2,244	89,668
**6**	196	153,452	708,740	487	2,387	89,807
**7**	322	153,664	708,406	221	2,872	90,293
**8**	279	154,100	708,013	3,371	3,092	90,514
**9**	205	154,375	707,811	10,565	6,621	94,050
**10**	222	154,698	707,473	639	17,179	104,607
**11**	268	154,920	707,207	4,060	17,884	105,321
**12**	532	155,184	706,680	406	21,940	109,367
**13**	202	155,716	706,479	3,366	22,336	109,762
**14**	414	156,046	705,936	159	25,700	113,113
**15**	1,108	156,454	704,843	3,883	25,857	113,261
**16**	585	157,561	704,260	162	54,865	177,382
**17**	253	158,141	704,013	139	54,957	647,939
**18**	637	158,392	703,379	171	78,225	409,425
**19**	2,444	159,024	700,935	302	172,510	681,489
**20**	2,045	161,607	698,753	151	309,545	309,848
**21**	2,622	163,649	696,135			
**22**	310	166,344	695,751			
**23**	420	166,714	695,271	**Long repeat sequences**		10,565 * 2 bp
**24**	6,180	167,250	688,964			
**25**	2,080	173,438	686,884	**Forward match (F)**		20 pairs
**26**	1,471	175,651	685,280			
**27**	2,834	177,197	682,379	**Palindromic match (P)**		30 pairs
**28**	397	330,626	501,796			
**29**	347	374,491	423,250			
**30**	302	681,489	689,582			

### 3.3 RNA-editing sites analysis

**Figure 2:**
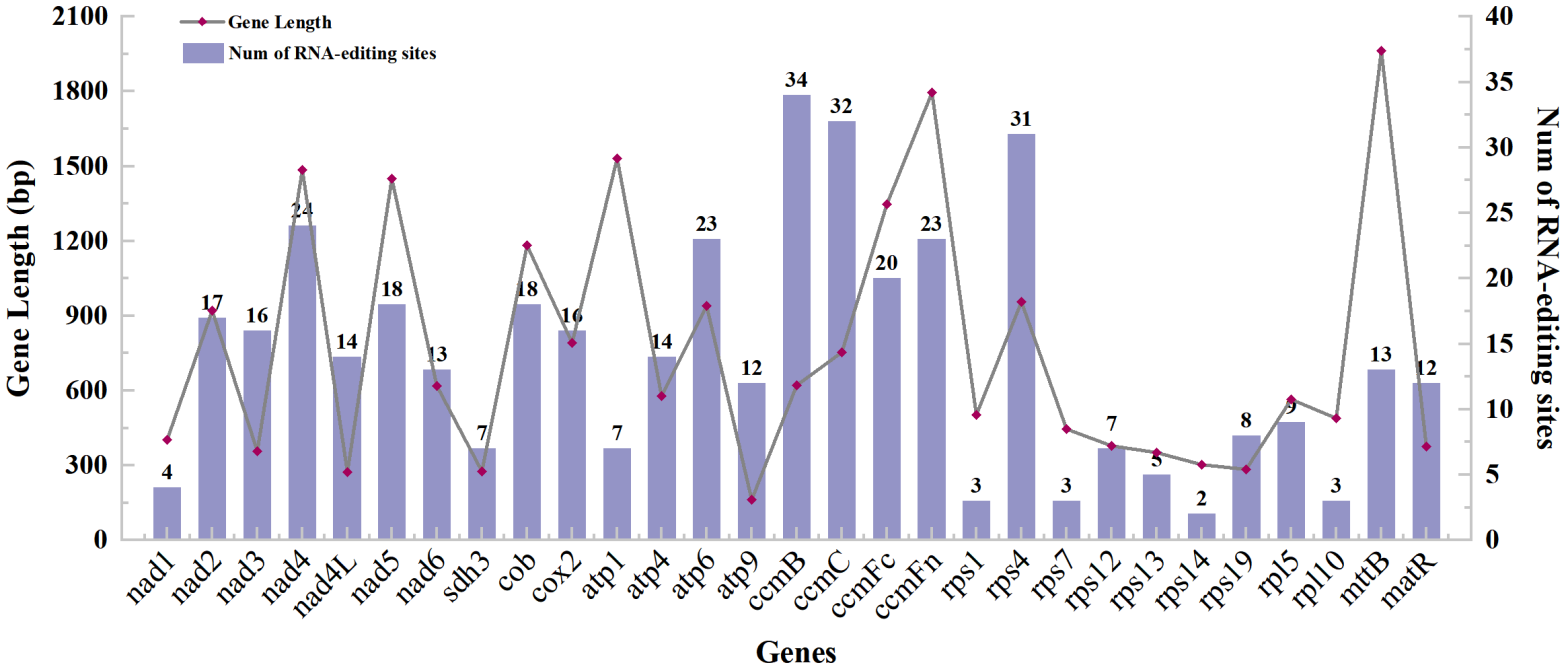
The gene length and predicted RNA-editing sites of protein-coding genes of the *Camellia oleifera* cv. Huashuo mt genome.

With 35 protein-coding genes (including multiple copies of genes), 413 RNA-editing sites were predicted in the mt genome of *Camellia oleifera*. The *ccmB*, which belongs to Cytochrome C biogenesis, had the most editing sites (34), while rps14 had the least (2) (**Supplementary Table 3**). By analyzing the relationship between the gene length and the number of RNA-editing sites, it was found that the longer the coding sequence, the more RNA-editing sites. However, there was no absolute linear relationship between them (**Figure 2**). All the RNA-editing were “C” to “U,” and the number of editing sites at the second base of codon was the highest. There were predicted 267 sites (64.65%) and 20 sites (4.84%) at second base and first and second base of codon, respectively. Furthermore, no site at third base of codon was predicted (**Supplementary Table 4**). Among all editing sites, 105 sites (25.42%) enabled serine to convert into leucine, and 89 sites (21.55%) enabled proline to convert into leucine. They account for almost half of the total. However, the conversion of two glutamine and two arginine to terminators was predicted (**Table 4**).

**Table 4:** The amino acid transformation of RNA-editing sites in the *Camellia oleifera* cv. Huashuo mt genomes.

Amino acid	A-V	H-Y	L-F	P-F	P-L	P-S	Q-X	R-C	R-X	R-W	S-F	S-L	T-I	T-M
**Count**	12	23	13	20	89	33	2	34	2	19	50	105	6	5
**Proportion(%)**	2.91	5.57	3.15	4.84	21.55	7.99	0.48	8.23	0.48	4.60	12.11	25.42	1.45	1.21
**Base position**	2	1	1	1/2	2	1	1	1	1	1	2	2	2	2

### 3.4 Phylogenetic analysis of mt genomes

**Figure 3:**
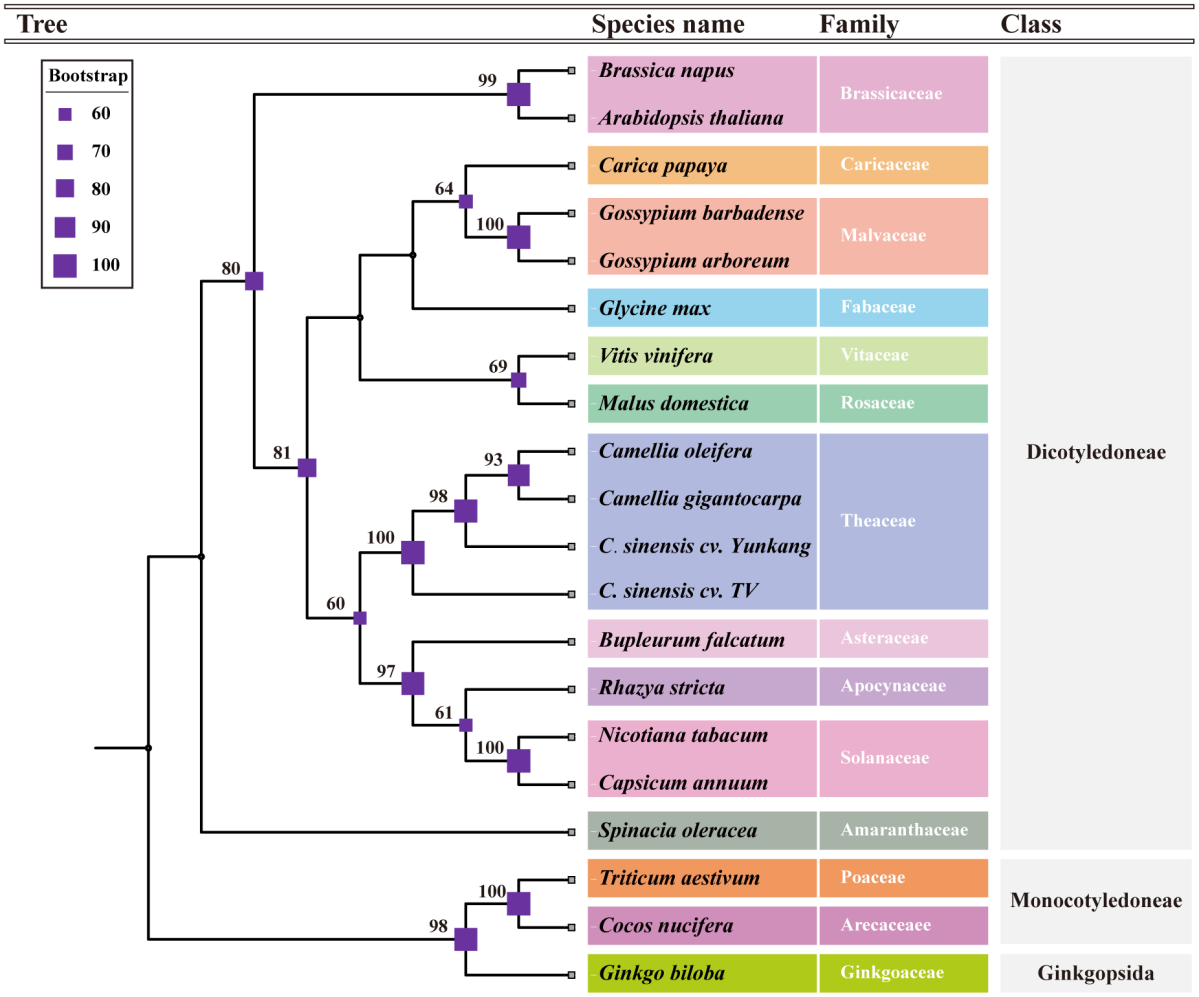
The phylogenetic tree based on the alignment of 18 other plants and *Camellia oleifera* cv. Huashuo mt genomes with bootstrap support values on each node. Different families can be distinguished by color.

A total of 15 conserved genes were found from 20 mt genomes, including the *C.oleifera* mt genomes (*atp1*, *atp6*, *atp9*, *ccmB*, *ccmC*, *ccmFc*, *ccmFN*, *cob*, *cox2*, *nad3*, *nad4*, *nad4L*, *rps12*, *rpl5*, and *matR*). The protein sequences of 15 genes were compared and then tandem connected to obtain the super-sequence gene file to construct the phylogenetic tree. The ModelFinder program of IQ-Tree software had tested 546 protein models, and HIVw+F+R3 was selected as the most suitable model. All species were clearly divided into three groups in the phylogenetic tree, including gymnosperm, monocotyledon, and dicotyledon, of which dicotyledon was the main group (**Figure 3**). *C. oleifera* with two cultivars of *C. sinensis* var. *assamica* (YK10 and TV-1) and *C. gigantocarpa* were grouped together in a branch with a bootstrap value of 100%. Species belonging to the same family were successfully grouped together.

### 3.5 Comparative analysis with *Camellia* species

**Figure 4:**
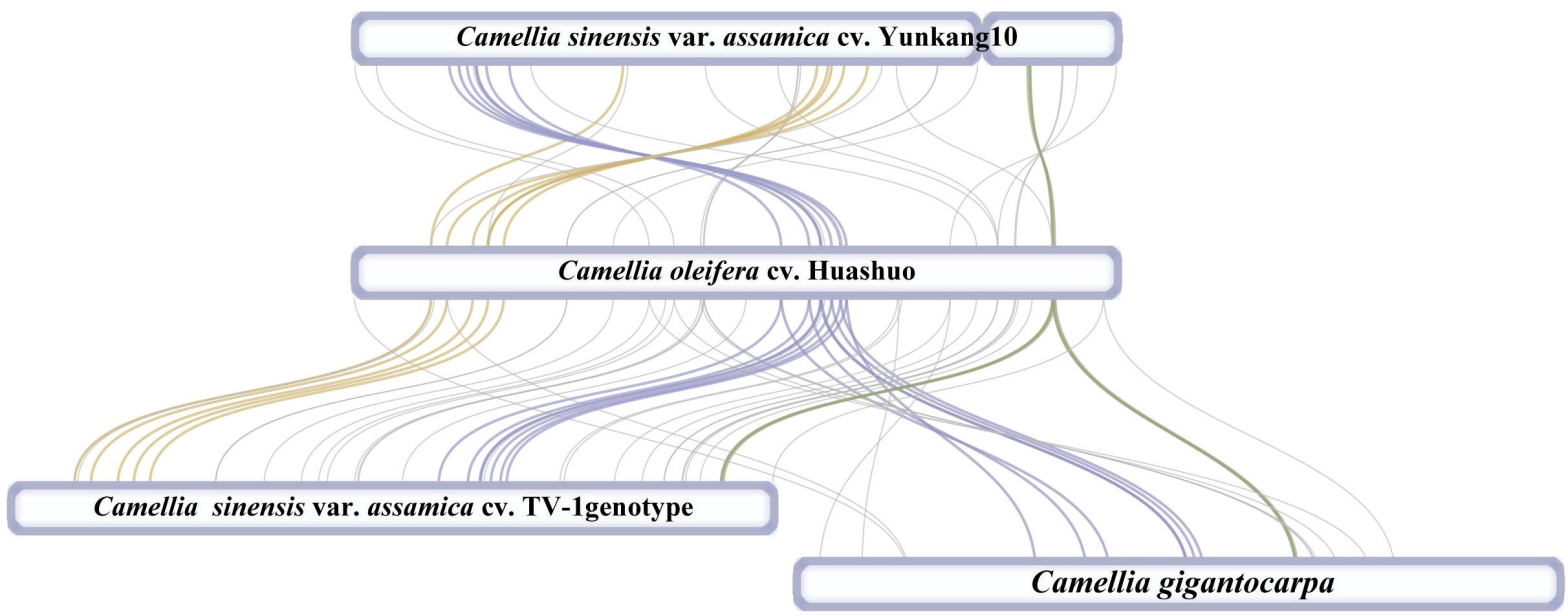
Collinearity analysis with mt genomes of four *Camellia* species. The genes indicated by the colors correspond to those in **Figure 4**.

**Figure 5:**
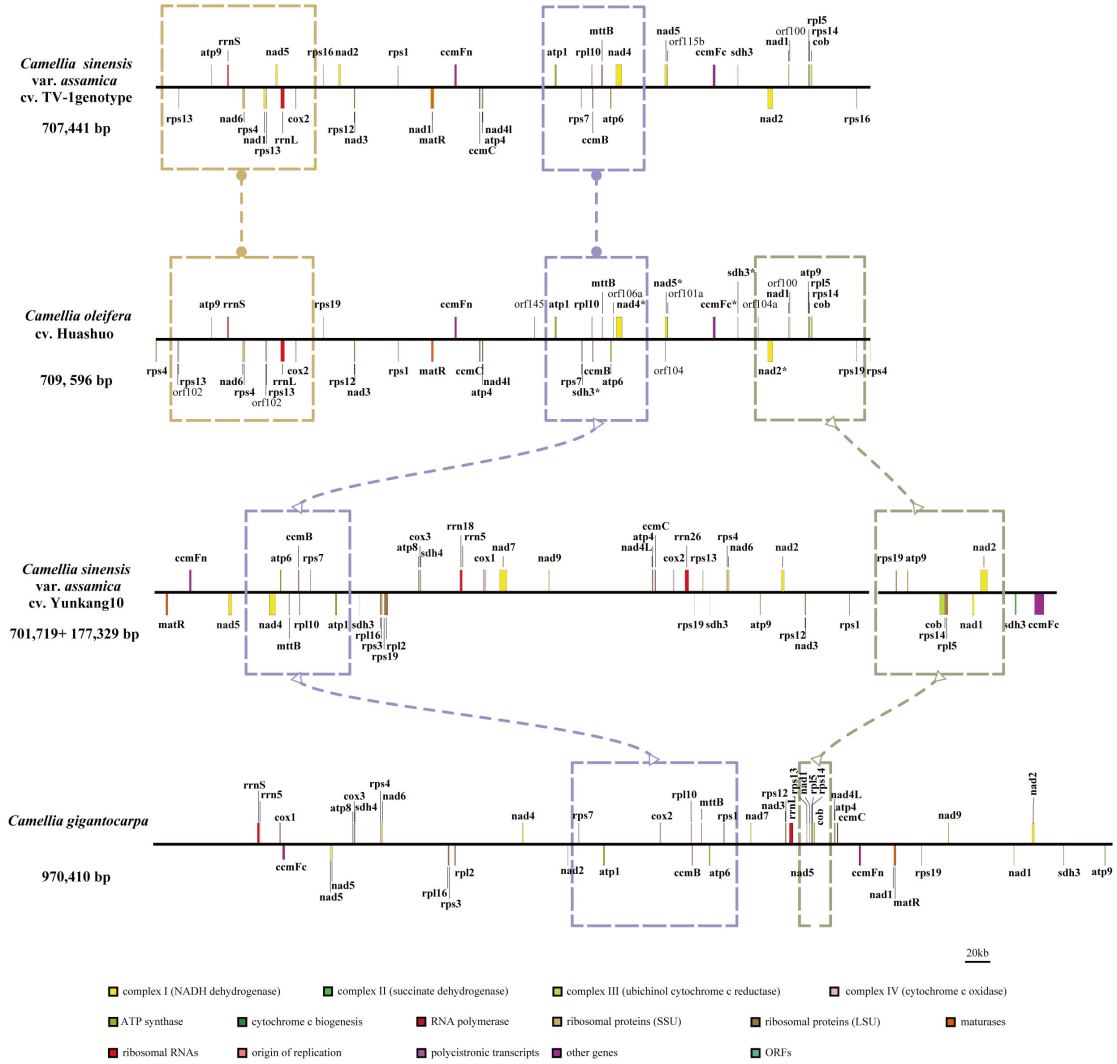
Comparison of mt genomes of four *Camellia* species (without transfer RNAs). Parts selected by the same color box have similar structure.

At present, only three mt genomes (*C. sinensis* var. *assamica* cv. TV-1, *C. sinensis* cv. Yunkang10, and *C. gigantocarpa*) had been reported in the *Camellia* genus and even the Theaceae family. The mt genome of TV-1 and *C. gigantocarpa* had one single circular map of 707,441 and 970,410 bp, and YK10 had two complete circular maps (701,719 and 177,329 bp). *C. gigantocarpa* has the largest mt genome, and *C. gigantocarpa* and YK10 have the largest number of protein-coding genes (44), followed by *C. oleifera*. It was found that the succinate dehydrogenase group of *C. oleifera* and YK10 had the same gene composition. The NADH dehydrogenase group and ribosomal protein group of *C. oleifera* and TV-1 have the same gene composition. By comparing linear maps of four mt genomes, it was found that the arrangements of some genes in all genomes were similar (**Figures 4**, **5**). The sites selected by the same color boxes in **Figure 4** had almost identical gene member, and the sequences of these genes in the four genomes were almost the same. The difference was that the genes were arranged on the sense strand of *C. oleifera*, *C. gigantocarpa*, and TV-1, but on the anti-sense strand of the YK10. For example, the composition of gene clusters *atp1*, *rps7*, *rpl10*, *ccmB*, *mttB*, *atp6* and *nad4* was relatively conserved, and their arrangement was the same on mt genomes of *C. oleifera* and TV-1, but reversed on YK10’s. The same was true of the other two gene clusters (*rps13*, *rrnL*, and *cox2*, and *rpl5*, *rps14*, and *cob*).

### 3.6 Conservation of tandem gene pairs on genomes

**Figure 6:**
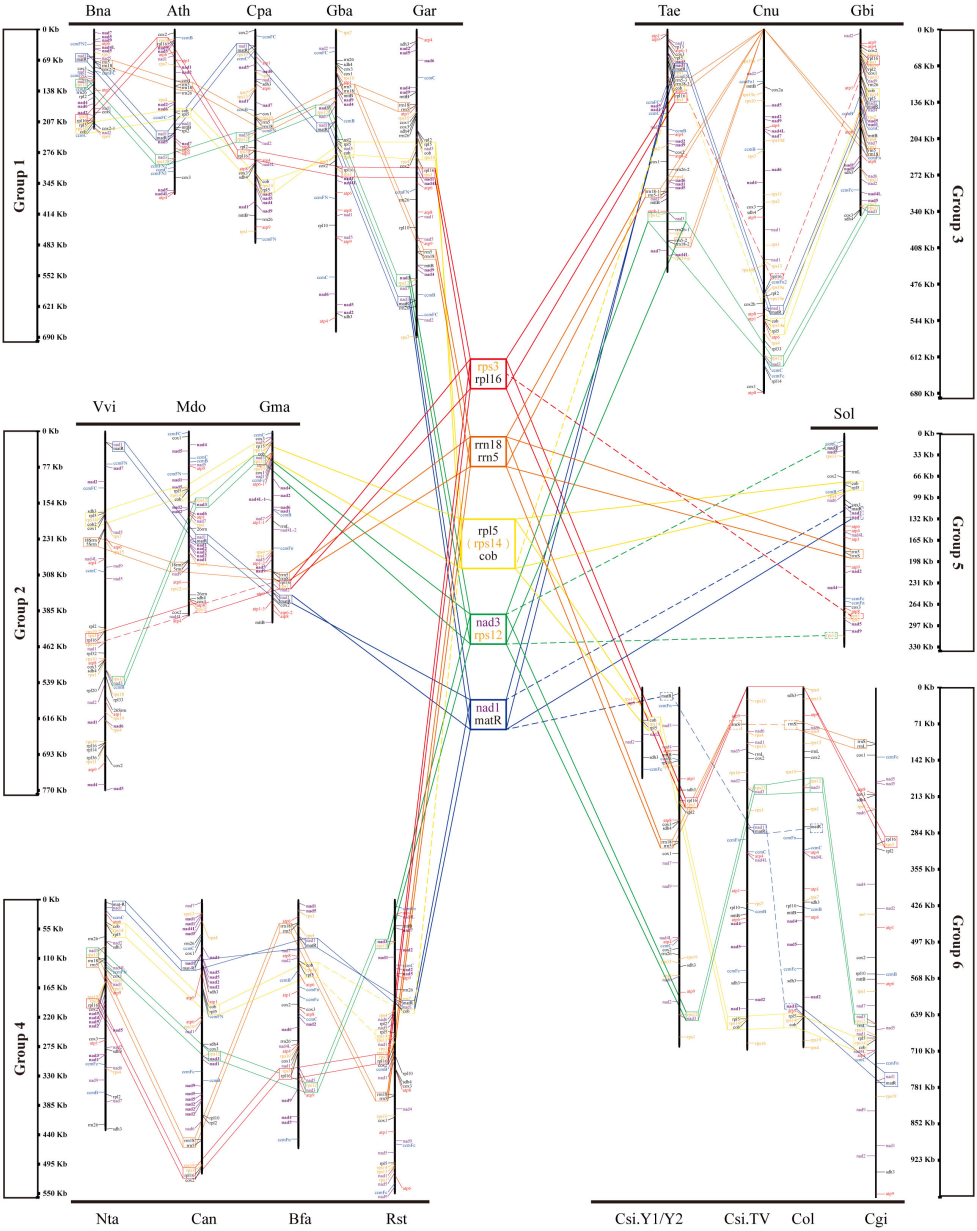
Gene localization in mt genomes of all species in the phylogenetic tree. Five gene clusters are in the middle of the figure.

To study the conserved gene clusters on the mt genomes of all species, including *Camellia*, we mapped the linear mt genome structure map of 20 species (**Figure 6**). We found 17 conserved genes on 20 mt genomes, including 15 protein-coding genes and two ribosomal RNAs genes (*rrnL* and *rrnS*). By analyzing the location information and upstream and downstream genes of these 17 genes on the 20 mt genomes, we did not find their arrangement rules on the genome. But back to the location information of all the genes on 20 mt genomes, we found five conserved tandem gene pairs from 20 species, namely, *rps3*-*rpl6*, *rrn18*-*rrn5*, *rpl5*-*cob*, *nad3*-*rps12*, and *nad1*-*matR*. Sometimes, *rpl5*-*rps14*-*cob* tandem gene pairs existed on the genome instead of *rpl5*-*cob*.

In all the mt genomes, tandem gene pairs may be located on the positive-sense strand or the antisense strand. The arrangement of some tandem gene pairs was reversed on some mt genomes. For example, on the mt genomes of Bna, Ath, Cpa, and Gba, *nad3*-*rps2* gene pairs located on the positive-sense strand, but on the anti-sense strand of Vvi, Mdo, and Gma mt genomes. The sequence of *nad1*–*matR* tandem gene pairs was inversely complementary on the Ath and TV-1 mt genomes. However, some genes in these five tandem gene pairs were missing on the genomes of individual species. For example, *rpl6* is missing on Mdo and Sol mt genomes, and *rrn5* is missing on TV-1 and Col mt genomes. These differences, such as the number and the type of genes annotated, may be related to the different annotation methods. Therefore, according to the arrangement of these five conserved gene pairs mentioned above, 20 species were divided into six groups. Bna, Ath, Cpa, Gba, and Gar formed group One. Vvi, Mdo, and Gma formed group Two. YK10, TV-1, Cgi, and Col were group Three. Rst, Bfa, Nta, and Can were group Four. Group Five only had Sol. Finally, Tae, Cnu, and Gbi formed group Six. The members of these six groups were consistent with the species branches in the evolutionary tree results. Therefore, these five gene pairs had a certain conservation in the evolutionary process.

### 3.7 Tandem rule of *nads* on mt genome

**Figure 7:**
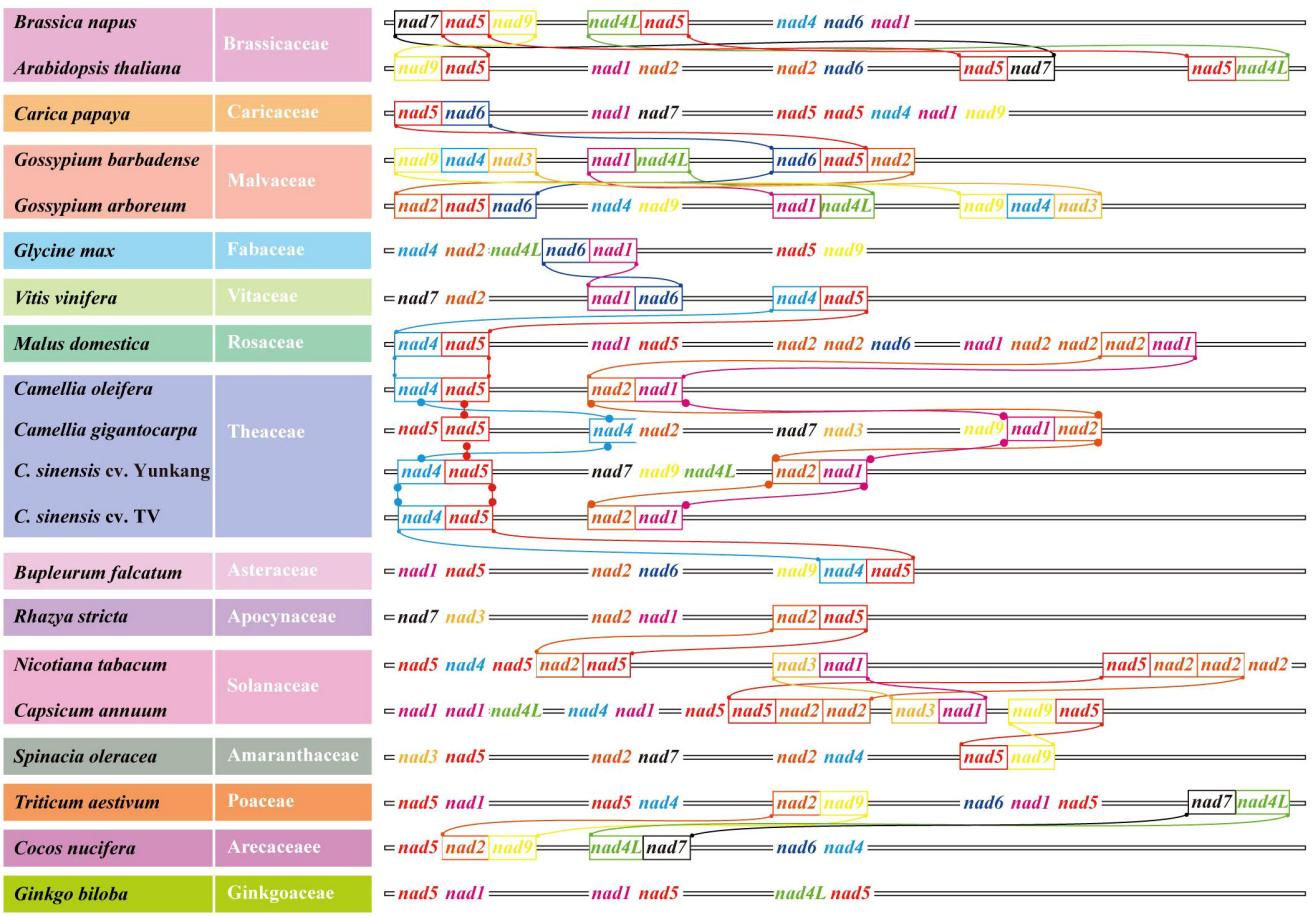
*Nad*–*nad* gene clusters in mt genomes of all species in the phylogenetic tree. Different *nad* genes were color coded. Identical tandem gene pairs between adjacent species were connected by curves.

**Figure 8:**
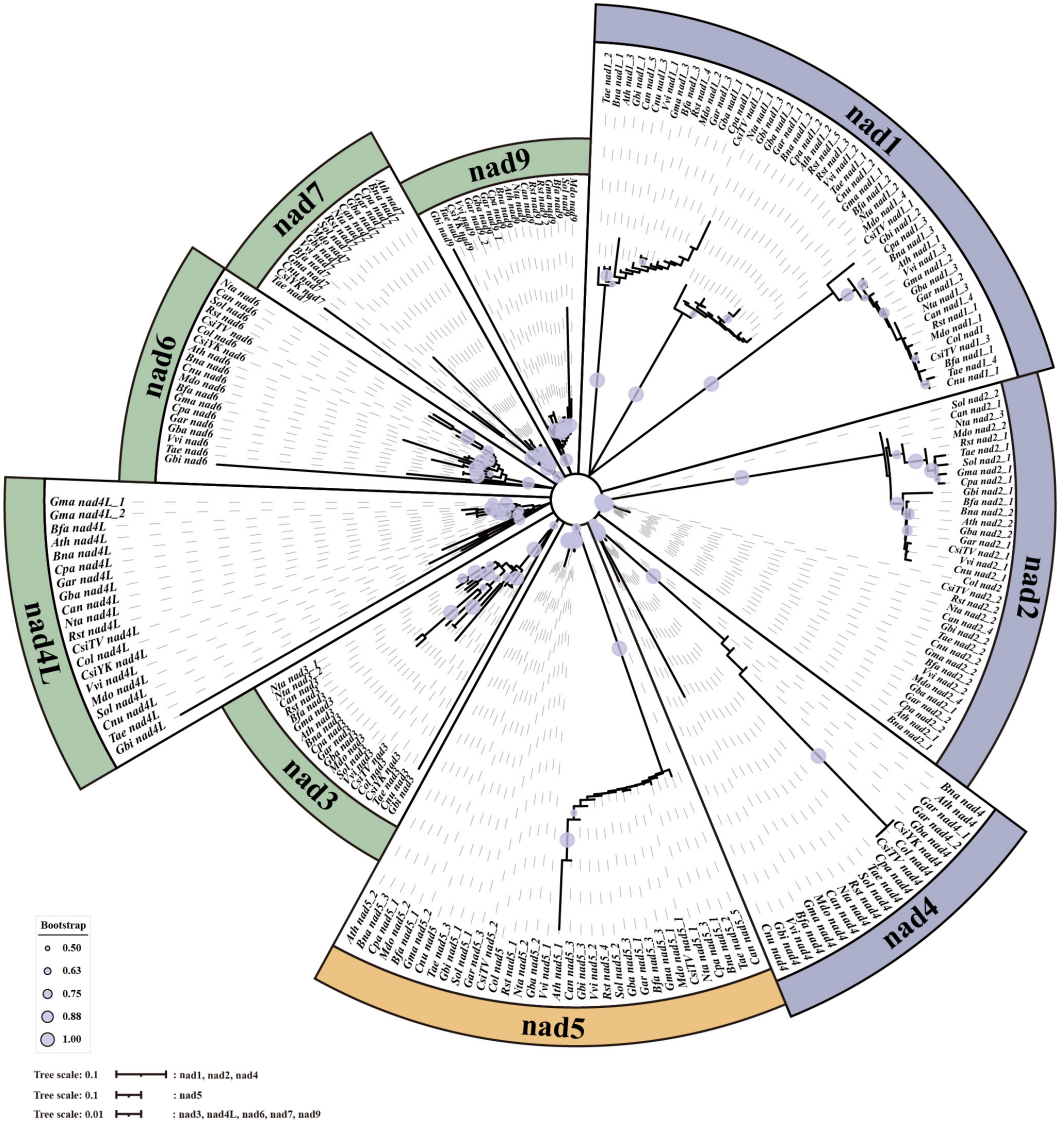
The phylogenetic tree of *nad* genes in mt genomes.

When analyzing the number and location information of all gene families on 20 genomes, several *nad*–*nad* gene pairs were found on each mt genome (**Figure 7**). For example, on Col, YK10, and TV-1, *nad4* and *nad5* were next to each other and also *nad2* and *nad1*. All *nads* with tandem arrangement in each species were identified, and the rules of *nad*–*nad* gene tandem were found in the six groups obtained from the previous classification. It was found that *nad1*, *nad5*, *nad6*, and *nad9* genes have the *nad*–*nad* gene tandem rule in group One, *nad1*, *nad2*, *nad4*, *nad5*, and *nad6* in group Two. In group Four, *nad1*, *nad2*, and *nad5* had the tandem rule and also *nad4L* and *nad5* in group Six. By integrating the results, it was found that all the mt genomes had the *nad5* gene, and there was also another *nad* gene next to it. So *nad5* always had the tandem rule during the evolution of the *nad* gene family. All the sequences of *nad* family genes were obtained from mt genomes, and the genes with a length less than 200 bp were removed. Finally, 233 *nad* genes were obtained for constructing the phylogenetic tree (**Figure 8**). Comparing the phylogenetic tree results of all nad subfamilies, the genetic distance of *nad5* was different with that of other subfamilies, which was divided into a separate group. At the same time, a species might have multiple *nad5* genes, and the *nad5* genes of all species were located in two branches of the evolutionary tree.

## 4 Discussion

The common *Camellia* oil tree known to us is a hexaploid plant with huge genomic data and complex structure (Gong et al., 2022). The chloroplast genome and mt genome could help to provide a more comprehensive auxiliary analysis of the genome evolution of *C. oleifera* (Li et al., 2021). With the advancement of sequencing methods, we have obtained more accurate sequences for the genome assembly. At the same time, original and simple methods are used to dig out the information from the genome sequence. So, this paper presents a complete mt genome of *C. oleifera* cv. Huashuo with a length of 709,596 bp, which is larger than most of the known plant mt genomes. Our lab has reported the chloroplast genome of *C. oleifera* cv. Huashuo (Wu et al., 2020), and the goal of this study is to analyze the mt genome of *C. oleifera* based on the sequencing data.

### 4.1 Differences in gene quantity and conservation of gene arrangement among *Camellia* species

Compared with mt genomes of *C. sinensis* var. *assamica* (YK10, Zhang et al., 2019 and TV-1 Rawal et al., 2020) and *C. gigantocarpa*, the mt genome size of *C. oleifera* was 709,596 bp, which was similar to the mt genome of TV-1 (707,441 bp) (**Table 1**). The mt genome of YK10 and *C. gigantocarpa* was longer than theirs. According to all the previous studies, it was found that the evolution of plant mt genome was more complicated than that of chloroplast genome. The genome size, composition, and gene sequence varied greatly among different species, among different individuals within the same species, and even among different cells of the same individual (Li et al., 2011). Moreover, the two cultivated tea plants are from China and India. But even then, like the Bryophyte (Liu et al., 2014), the mitochondria genome remained structurally stable through evolution. Thus, all plants cannot be generalized; they need to have a case-by-case analysis.

A total of 35 protein-coding genes, 29 tRNAs, and 2 rRNAs were annotated in the mt genome of *C. oleifera*. The protein-encoding genes of TV-1 were four fewer than that of *C. oleifera*, and the total number of genes was four fewer than that of *C. oleifera*. The sequenced mt genomes of plants have been annotated to obtain approximately 35 protein-coding genes, such as 35 in *Vitis vinifera* (Goremykin et al., 2009), 36 in *Leucaena Trichandra* (Kovar et al., 2018), and 33 in *Dalbergia odorifera* (Hong et al., 2021). YK10 and Cgi has 9 protein-coding genes more than *C. oleifera*, but 5 and 10 tRNA genes fewer than the other two. Unseld et al. (1997) found that the number of genes was different among different species, which might be related to the number of ribosomal subunit genes and tRNAs (Li et al., 2011). Indeed, rpl genes in the *C. oleifera* mt genome (2 genes) and TV-1 (2 genes) are less than those in YK10 and Cgi (4 genes). In addition, the NADH dehydrogenase gene of *C.oleifera* was also lost compared with YK10 and Cgi. *C. oleifera*, Cgi, YK10, and TV-1 all belong to the Theaceae, but there is a great quantity variance in the number of annotated genes. It is possible that the methods used in genome annotation made this situation in closely related species. It is worth paying attention to the occurrence of such cases with comparative genomic analysis in the future. The mt of *C. oleifera* also contained eight ORF genes, which have been found in previous studies to be usually related to the “infertility” problem in plants (Chaumont et al., 1995). For example, the *T2urf13* in maize, *orf79* gene in rice, and *orf224*/*orf138* in rape are all associated with cytoplasmic male sterility (Duroc et al., 2005). The function of rich *orf* genes in *C. oleifera* mt can be explored later in combination with plant characteristics and nuclear genes.

RNA-editing sites can affect gene function by changing the protein folding pattern. The genome size and number of protein-coding genes of *C. oleifera* were larger than that of TV-1, but there were fewer RNA-editing sites. The number of protein-coding genes of *C. oleifera* and YKg10 differed by 9, but 65 differences in RNA-editing sites. These results confirmed that an increase in the number of bases in the mt genome did not lead to an increase in RNA-editing sites. At the same time, the number of editing sites of the same gene in different species is also different. In addition, when studying early terrestrial plants, Zhang found that the number of RNA-editing sites ranged from 0 to 2,152, and different types and numbers of RNA-editing sites contributed to the characteristics of plants (Zhang et al., 2020).

### 4.2 Conserved gene clusters exist during the evolution of species, especially *nad5*

From the mt genome of *C. sinensis* and other previous studies, it is found that the species can be correctly classified by constructing an evolutionary tree through conserved genes. In this study, we screened 15 conserved genes from 20 mt genomes, and finally *C. oleifera*, *C. sinensis*, and *C. gigantocarpa* clustered together in one branch, and other species belonging to the same family also clustered together. We found that the number of conserved genes screened for evolutionary tree construction accounted for about one-third of the total number of genes (excluding tRNA genes). Concurrently, when comparing four mt genomes of *Camellia*, we also found that there were structurally conserved regions in the sequences of *C. oleifera* and other three.

Rearranging the position of genes in the mt genome can occur during evolution as the result of sequence break and sequence recombination. However, some highly conserved genes or gene clusters maintained their original evolutionary patterns throughout the process of evolution (Niu et al., 2022). In this study, five conserved gene clusters were identified across the 20 species: *rps3*-*rpl6*, *rrn18*-*rrn5*, *rpl5*-*cob*, *nad3*-*rps12*, and *nad1*-*matR*. But it was observed that some gene of these five gene pairs also were absent in some species. For instance, this study found that *S. oleracea* lacks the *rpl16* gene, which is also missing in *Melastoma dodecandrum* (Cai et al., 2017; Zhou et al., 2023). Additionally, *C. nucifera* lacks the *rps3*, which was relatively conserved in all species studied. So, this study grouped all species according to the conservativeness of all genes and found that the results of the grouping matched the position on the phylogenetic tree. Therefore, the absence of genes can be used to distinguish different evolutionary groups of species. The conservativeness of gene pairs can also be used to analyze the distance of genome evolution. Niu et al. found two conserved gene clusters (*rps12*-*nad3* and *rps3*-*rpl16*) in eight mt genomes (Niu et al., 2022). This result overlaps with the gene pairs found in this study, which shows that the conservativeness of gene clusters is universal in different species. It can be further explored during the study of mt genome.

When analyzing conservative genes, it was found that the *nad* gene has its own genetic rule. The *nad* gene is a part of the large enzyme complex of complex I, and it is active in the mitochondria (Wu et al., 2022). Complex I is one of the enzyme complexes necessary for oxidation phosphorylation. The mitochondrion is the main organelle that produces energy in the cell, which produces adenosine triphosphate (ATP), the main energy source of the cell, through the process of oxidative phosphorylation (Gualberto et al., 2013; Zhao et al., 2023). Therefore, for the whole life of an organism, the *nad* gene is a key role. When analyzing the genetic inheritance rules between different species, it showed that *nad* gene tandem arranging was common in all the mt genomes. For example, *nad5*–*nad4* in *C. oleifera* and *C. sinensis*, *nad5*–*nad9*, *nad1*–*nad2*, and *nad5*–*nad7* in *A. thaliana* were located in adjacent locations in the mt genome (Unseld et al., 1997). With the phylogenetic tree, there were common *nad* tandem pairs in the species with close parental relationship. For example, *nad1/2/3/4/5/9/4L* in *G. barbadense* and *G. arboretum* has genomic tandem (Tang et al., 2015). Also, *nad1/2/3/4/5* in the mt genomes of *N. tabacum* and *C. annuum* existed in tandem (Sugiyama et al., 2005; Magee et al., 2010). In the mt genomes of gymnosperms, such as *G. biloba*, all *nad–nad* tandem gene pairs contain *nad5* (*nad5*–*nad1* and *nad5*–*nad4L*). It was found that, except for *Oryza minuta*, the mt genomes of most species contain *nad5* (Sajjad et al., 2016; Niu et al., 2022), and the *nad5* genes were arranged in tandem. Similarly, in the mt genome of *Dalbergia odorifera*, the *nad5* gene was found to be linked in tandem with *nad1*, as was the case with the *nad5* gene in *Leucaena trichandra* (Kovar et al., 2018; Zhou et al., 2021). Therefore, other *nad* genes may be required to induce or enhance the function of *nad5* in mitochondria life activity. *nad5* may have an ability to enhance its tandem genes’ function.

### 4.3 Software parameters on gene annotation in mt genomes

When we annotated the *Camellia oleifera* mitochondrial genome, we found that the gene number of *nads* and other gene families was correlated with the software parameters that we set. At first, when the annotated genes were screened, the “coverage” and “match” were set to more than 40%. Twelve *nad* genes and all 48 protein-coding genes were obtained (**Supplementary Figure 1**). But when the two parameters were increased to 60% or even 90%, only seven *nad* genes were annotated (**Supplementary Figure 1**). In our manuscript, parameters with 60% were used. It was also found when we reannotated the mitochondrial genome of TV-1. In addition, the evolution-related regularities we found in our manuscript were mainly related to these seven *nad* genes. Combined with the subsequent analysis of the paper, we found that adjusting the values of coverage and matching degree during screening could help in obtaining more accurate results in comparative genomic analysis.

## 5 Conclusion

In summary, by sequencing and analyzing the mt genome of *Camellia oleifera*, we found the general tandem array of the *nad* gene family in the mt genome. Through the analysis of *nad* gene families in different species, we found that the *nad* gene with tandem relationship was species specific. In addition, it was noteworthy that the *nad5* has tandem gene pairs of *nad5*–*nads* in all species. The discovery of conserved genes and conserved gene pairs will provide a new direction for the study of the mitochondria.

## Data Availability

The data that support the findings of this study have been deposited into CNGB Sequence Archive (CNSA) of China National GeneBank DataBase (CNGBdb) with accession number CNP0005996.

## Supplementary material

Supplementary Figure 1 Gene annotation results with different parameters.

Supplementary Table 1 SSR motifs annotation of *C. oleifera* cv. Huashuo mt genomes.

Supplementary Table 2 Long repeat sequences annotation of *C. oleifera* cv. Huashuo mt genomes.

Supplementary Table 3 Statistics of RNA-editing sites in the *Camellia oleifera* cv. Huashuo mt genomes.

Supplementary Table 4 RNA-editing sites annotation of *C. oleifera* cv. Huashuo mt genomes.
