# Supplementary figures and images for: Mitochondrial genome study of Camellia oleifera revealed the tandem conserved gene cluster of nad5–nads in evolution
Source: Front Plant Sci. 2024 Sep 3;15:1396635. doi: 10.3389/fpls.2024.1396635 (PMC11405228; doi:10.3389/fpls.2024.1396635)

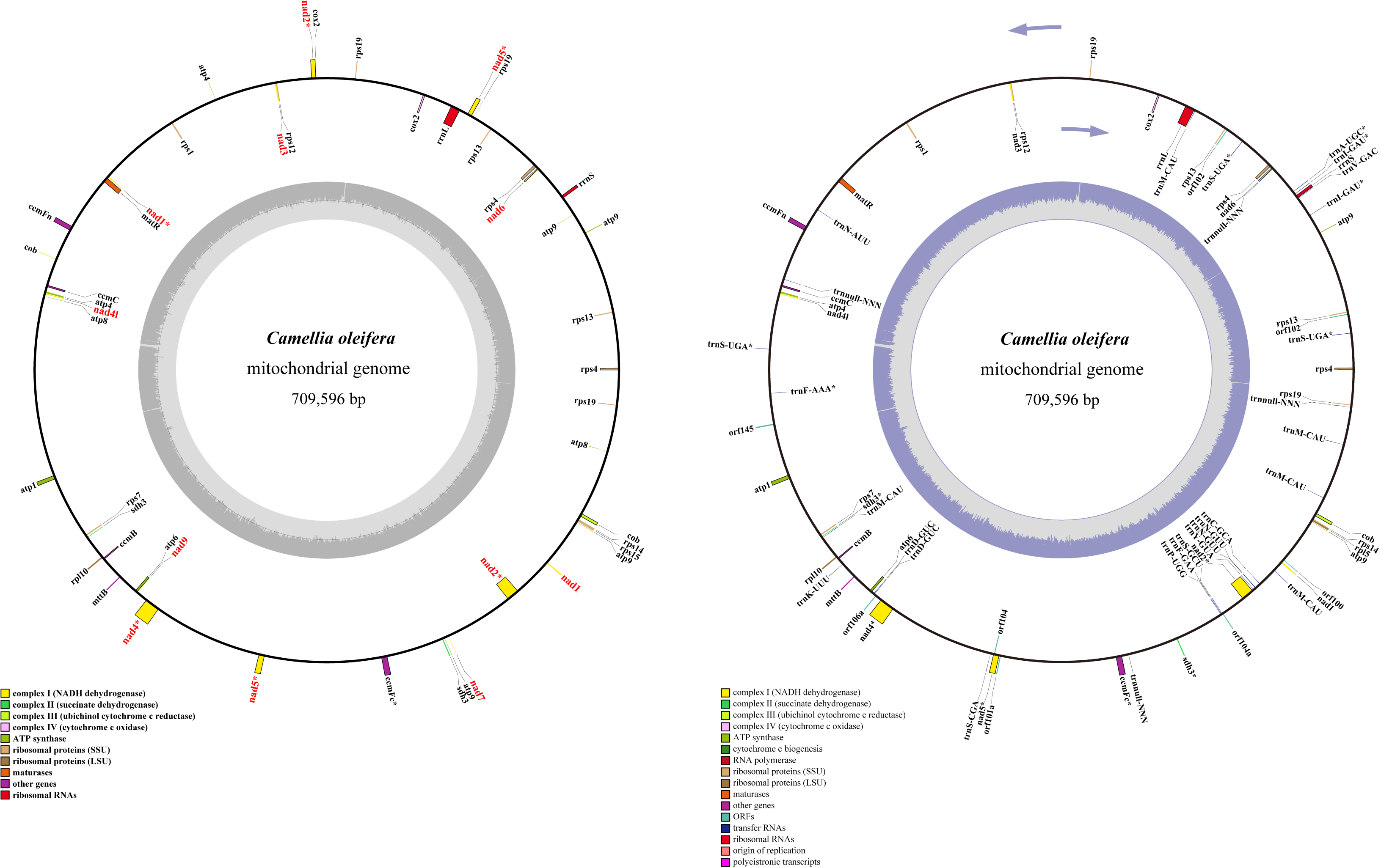

Supplement: Supplementary Figure 1 — Gene annotation results with different parameters. [file Image1.tif]
